# Determinants of Perivascular Spaces in the General Population: A Pooled Cohort Analysis of Individual Participant Data

**DOI:** 10.1212/WNL.0000000000201349

**Published:** 2023-01-10

**Authors:** Tavia E. Evans, Maria J. Knol, Petra Schwingenschuh, Katharina Wittfeld, Saima Hilal, M. Arfan Ikram, Florian Dubost, Kimberlin M. H. van Wijnen, Petra Katschnig, Pinar Yilmaz, Marleen de Bruijne, Mohamad Habes, Christopher Chen, Sönke Langer, Henry Völzke, M. Kamran Ikram, Hans J. Grabe, Reinhold Schmidt, Hieab H.H. Adams, Meike W. Vernooij

**Affiliations:** From the Departments of Clinical Genetics (T.E.E., M.J.K., H.H.H.A.), Radiology and Nuclear Medicine (T.E.E., F.D., K.M.H.W., P.Y., M.B., H.H.H.A., M.W.V.), Epidemiology (M.J.K., M.A.I., P.Y., M.K.I., M.W.V.), and Neurology (M.K.I.), Erasmus MC, Rotterdam, the Netherlands; Department of Neurology (P.S., P.K., R.S.), Medical University of Graz, Austria; German Center for Neurodegenerative Diseases (DZNE) (K.W., M.H., H.J.G.), Site Rostock/Greifswald; Department of Psychiatry and Psychotherapy (K.W., H.J.G.) and Institute of Diagnostic Radiology and Neuroradiology (S.L.), University Medicine Greifswald, Germany; Department of Pharmacology (S.H., C.C.), National University of Singapore; Memory Aging & Cognition Centre (MACC) (S.H., C.C., M.K.I.), National University Health System, Singapore; Saw Swee Hock School of Public Health (S.H.), National University of Singapore; Department of Biomedical Data Sciences (F.D.), Stanford University, CA; J. Philip Kistler Stroke Research Center (P.Y.), Department of Neurology, Massachusetts General Hospital, Harvard Medical School, Boston; The Machine Learning Section (M.B.), Department of Computer Science, University of Copenhagen, Denmark; Neuroimage Analytics Laboratory (NAL) and the Biggs Institute Neuroimaging Core (BINC) (M.H.), Glenn Biggs Institute for Alzheimer's and Neurodegenerative Diseases, University of Texas Health Science Center San Antonio (UTHSCSA), TX; and Latin American Brain Health (BrainLat) (H.H.H.A.), Universidad Adolfo Ibáñez, Santiago, Chile.

## Abstract

**Background and Objectives:**

Perivascular spaces (PVS) are emerging markers of cerebral small vessel disease (CSVD), but research on their determinants has been hampered by conflicting results from small single studies using heterogeneous rating methods. In this study, we therefore aimed to identify determinants of PVS burden in a pooled analysis of multiple cohort studies using 1 harmonized PVS rating method.

**Methods:**

Individuals from 10 population-based cohort studies with adult participants from the Uniform Neuro-Imaging of Virchow-Robin Spaces Enlargement consortium and the UK Biobank were included. On MRI scans, we counted PVS in 4 brain regions (mesencephalon, hippocampus, basal ganglia, and centrum semiovale) according to a uniform and validated rating protocol, both manually and automated using a deep learning algorithm. As potential determinants, we considered demographics, cardiovascular risk factors, *APOE* genotypes, and other imaging markers of CSVD. Negative binomial regression models were used to examine the association between these determinants and PVS counts.

**Results:**

In total, 39,976 individuals were included (age range 20–96 years). The average count of PVS in the 4 regions increased from the age 20 years (0–1 PVS) to 90 years (2–7 PVS). Men had more mesencephalic PVS (OR [95% CI] = 1.13 [1.08–1.18] compared with women), but less hippocampal PVS (0.82 [0.81–0.83]). Higher blood pressure, particularly diastolic pressure, was associated with more PVS in all regions (ORs between 1.04–1.05). Hippocampal PVS showed higher counts with higher high-density lipoprotein cholesterol levels (1.02 [1.01–1.02]), glucose levels (1.02 [1.01–1.03]), and *APOE* ε4-alleles (1.02 [1.01–1.04]). Furthermore, white matter hyperintensity volume and presence of lacunes were associated with PVS in multiple regions, but most strongly with the basal ganglia (1.13 [1.12–1.14] and 1.10 [1.09–1.12], respectively).

**Discussion:**

Various factors are associated with the burden of PVS, in part regionally specific, which points toward a multifactorial origin beyond what can be expected from PVS-related risk factor profiles. This study highlights the power of collaborative efforts in population neuroimaging research.

Perivascular spaces (PVS) are fluid-filled spaces encapsulating penetrating brain vessels. PVS are suggested to be vital for extracellular waste removal within the movement and draining of fluid in the brain. These PVS can dilate so that they become visible on MRI as spaces with signal intensities similar to that of CSF. Such PVS can occur throughout the brain but are more often seen in the white matter (WM) and deep gray matter.^1^ These regional differences are believed to be partly due to morphological factors, such as regional differences in the composition of membranes enclosing PVS,^2^ and the branching and caliber changes of penetrating vessels.^3^ Besides these morphological factors, however, it is believed that PVS in various locations might reflect different etiologies.

Although PVS were originally believed to be an insignificant finding, they have more recently been linked to normal aging^4^ but also to neurologic disorders, including stroke, cerebral small vessel disease (CSVD),^5-9^ Alzheimer disease,^10,11^ migraine,^12^ and multiple sclerosis.^13,14^ Reflecting the variety of associated diseases, studies have emerged on a broad range of PVS determinants. A major focus has been on aging, cardiovascular risk factors, and MRI markers of CSVD.^1,3,5,8,15-22^ In addition, some studies have investigated the relation with inflammation markers, cerebral amyloid angiopathy, and CSF biomarkers.^6,23,24^ Recent exploration into the genomics of PVS has also unveiled interesting insight into regional variabilities and genetic overlap with neurodegenerative diseases.^25,26^ However, previous studies have used heterogeneous methods to assess PVS, combined with often small samples, resulting in conflicting findings that are difficult to interpret. Furthermore, most studies only reported on 1 or 2 regions, mainly the basal ganglia and WM. The mesencephalon and, to a lesser extent, the hippocampus are generally absent from rating scales despite frequently containing PVS. Finally, most research has not compared results across different ethnicities.

Here, we investigated potential determinants of PVS in 4 brain regions, namely mesencephalon, hippocampus, basal ganglia, and centrum semiovale. We performed a pooled analysis of 10 population-based cohort studies with almost 40,000 individuals, all applying a uniform and validated rating method or an automated detection algorithm based on the aforementioned rating method,^27-29^ and examined associations of demographic factors, cardiovascular risk factors, *APOE* genotypes, and MRI markers with region-specific PVS burden. Furthermore, we performed stratification on self-reported ethnicity to perform an initial exploration into whether these associations differed across ethnic groups. We hypothesized differential associations between potential PVS determinants and PVS counts across different brain regions.

## Methods

**Figure 1:**
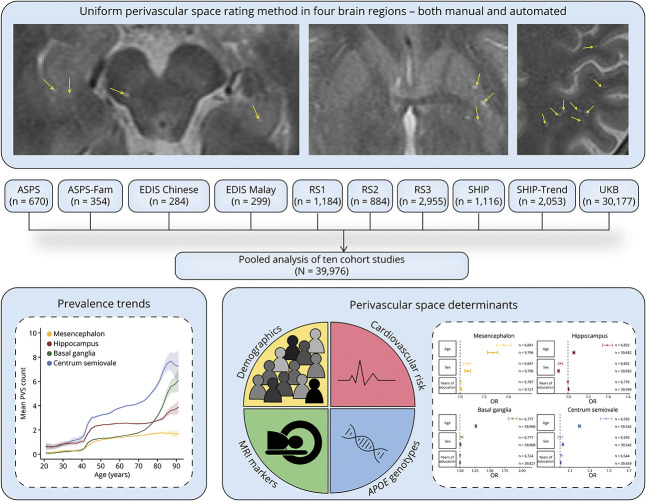
Overview Figure Showing the Design of This Study Abbreviations: ASPS = Austrian Stroke Prevention Study; ASPS-Fam = Austrian Stroke Prevention Family Study; EDIS = Epidemiology of Dementia in Singapore; RS = Rotterdam Study; SHIP = Study of Health in Pomerania; UKB = UK Biobank.

A schematic overview of this study is presented in Figure 1.

### Study Population

This study was performed as part of the Uniform Neuro-Imaging of Virchow-Robin Spaces Enlargement consortium, a collaboration between population-based cohort studies, complemented by the UK Biobank (UKB).^30,31^ This study included subjects from the Austrian Stroke Prevention Study (ASPS), the ASPS Family study (ASPS-Family), the Epidemiology of Dementia in Singapore study (EDIS Chinese and EDIS Malay), the Rotterdam Study (RS1, RS2, and RS3),^32,33^ the Study of Health in Pomerania study (SHIP and SHIP-Trend), and the UKB.^31^ More detailed information on these studies is presented in Table 1 and eTable 1 (links.lww.com/WNL/C400).

**Table 1:**
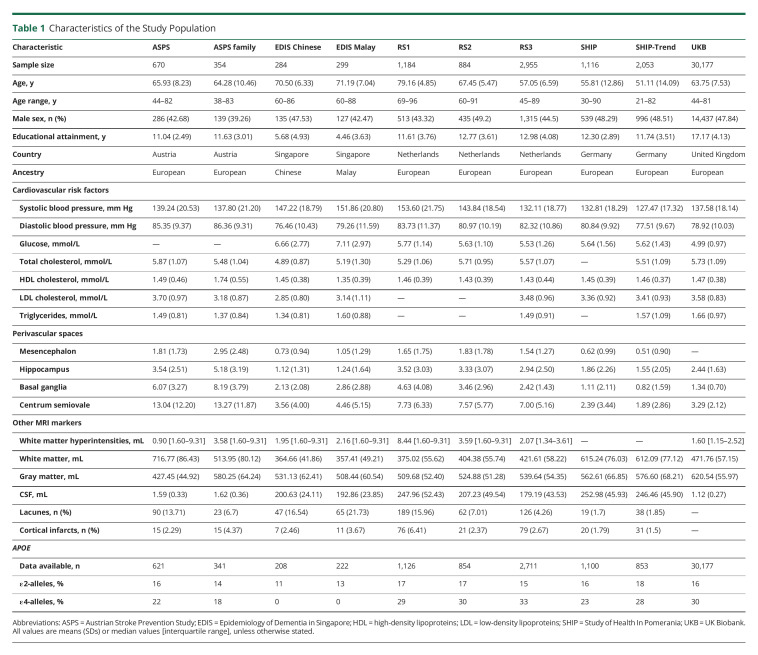
Characteristics of the Study Population

### Standard Protocol Approvals, Registrations, and Patient Consents

The individual studies have been approved by their local institutional review boards or ethics committees (Supplementary Information). Written informed consent was obtained from all participants.

### Image Acquisition

Various MRI scanners and protocols were used to acquire images, as previously described.^30,34^ In brief, the MRI field strength in ASPS-family, EDIS, and UKB was 3T, whereas all other studies used 1.5T. All studies had T1-weighted, T2-weighted, and fluid-attenuated inversion recovery (FLAIR) sequences, except for the UKB, SHIP, and SHIP-Trend, which did not have T2-weighted sequences available. The slice thickness of the primary rating sequence ranged between 1.0 and 5.5 mm. More detailed information on MRI scanners and protocols is presented in eTable 2 (links.lww.com/WNL/C400).

### PVS Rating

The PVS rating was performed in all cohorts according to 1 validated standardized manual rating method or an automatic extraction method based on this (RS3 and UKB).^27-29,35^ The primary rating sequence was the T2-weighted sequence for all studies except SHIP, SHIP-Trend, and UKB, in which T1-weighted sequences were used.

For the manual measurements, we have previously examined the effect of using T1-weighted images as the primary rating sequence and found a high reliability with using T2-weighted images (mean intraclass correlation coefficient [ICC] = 0.8).^30^ We counted PVS between 1 and 3 mm. The rating was performed in 4 brain regions: mesencephalon, hippocampus, basal ganglia, and centrum semiovale. For the latter 2 regions, which are large and can harbor hundreds of PVS, only a single slice was rated. For the basal ganglia, this was at the height of the anterior commissure, and for the centrum semiovale, this was 10 mm above the ventricles. Trained investigators rated PVS in ASPS, ASPS-Family (C.G., P.K., P.S., R.S., and T.P.), EDIS (S.H.), RS1, RS2, SHIP, and SHIP-Trend (H.H.H.A.) at each participating center, with good to excellent interrater and intrarater reliability, ICC 0.62–0.82 and >0.8, respectively.^27^ The size and shape of lesions, as well as the presence of a hyperintense rim on FLAIR images, were used to differentiate PVS from lacunes.

The automated method was trained using the abovementioned manual ratings within RS1 and RS2. This method is applied per region and provides the PVS count for that region.^28,29^ Initially, this method was developed and tested using T2-weighted sequences, obtaining ICCs above 0.8 for all regions.^28^ Later, the method was trained and tested using T1-weighted images (mean squared error difference from T2 method; 0.5–5). When applied to the UKB, the T1-weighted method produced unreliable results for the mesencephalon, leading to the exclusion of this region. Full details pertaining to the automated methodology have been thoroughly described before.^28,29^

### Assessment of Determinants

We investigated a range of potential determinants, including demographics, cardiovascular risk factors, *APOE* genotypes, and MRI markers. These determinants were chosen because of their suggested association with PVS in previous studies.^8^ We also included risk factors for other CSVD markers because of their known overlap with PVS.^22^

We first looked at age at the time of scanning, sex, and educational attainment. Cohort-specific education categories were recoded to years of education to make comparisons possible. We measured systolic and diastolic blood pressure (BP) and calculated pulse pressure, the difference between the 2. Blood samples were used to measure the levels of total cholesterol, high-density lipoprotein (HDL) cholesterol, low-density lipoprotein cholesterol, triglycerides, and glucose. Persons were coded according to their smoking status as never, former, or current smokers. *APOE* genotyping was performed using TaqMan assays, except for SHIP, SHIP-Trend, and UKB, where it was imputed from genotyping assays. MRI markers that were investigated included lacunes of presumed vascular origin and cortical infarcts, which were rated according to the established criteria.^36^ Tissue volumes were automatically determined using several segmentation algorithms.^37-40^ For this, voxels were classified as gray matter, WM, WM hyperintensities (WMH), and CSF, and all voxels in a single class were summed to obtain volumes. Intracranial volume (ICV) was the total of these volumes. Given differences in segmentation methods, all volumes were first standardized within cohorts and subsequently pooled. The UKB was additionally stratified using self-reported ethnicity (European, Asian, and African) coded by data field 21,000 (White, Asian or Asian British and Chinese, and Black or Black British, respectively).

### Statistical Analyses

PVS counts per region were analyzed as dependent variables with zero-inflated negative binomial regression models, taking into account their discrete nature and excess zeros using a probability distribution. Each of the determinants was modeled as an independent variable along with other covariates. To aid comparison, all determinants were standardized except age, sex, years of education, smoking status, presence of cortical infarcts and lacunes, and *APOE* genotypes. All analyses were adjusted for age and sex and volumetric measures additionally for ICV. Cardiovascular analyses were further adjusted for systolic and diastolic BP, cholesterol, body mass index (BMI), and glucose. Furthermore, we explored whether cardiovascular risk factors (i.e. systolic and diastolic BP, cholesterol, BMI, and glucose) and MRI markers (i.e. ICV, lacunes, cortical infarcts, and WMH volume) were independently associated with PVS counts by modeling them together. Random effects for cohorts were incorporated in the models. To take into account the number of potential determinants tested in this study, we also indicate which variables survive Bonferroni correction for multiple testing, corresponding to *p* < 0.0026 (0.05/19 variables). Analysis within SHIP-Trend and EDIS Chinese data sets did not consistently fit the negative binomial regression model, predominantly within the mesencephalon; thus, these analyses were omitted. These data were however included in the full pooled analysis. All analyses were performed in R (version 3.4.1) using the “glmmADMB” package.

### Data Availability

The data within this study are available either within the article and the supplementary material or from the authors on reasonable request.

### Standard Protocol Approvals, Registrations, and Patient Consents

ASPS was approved by the institutional review board of the Medical University of Graz. EDIS was approved by the relevant institutional ethics review boards (National Healthcare Group Domain Specific Review Board and the SingHealth Centralised Institutional Review Board). The RS has been approved by the Institutional Review Board (Medical Ethics Committee) of the Erasmus Medical Center and by the review board of the Netherlands Ministry of Health, Welfare, and Sports. SHIP was approved by the ethics committee of the University of Greifswald. UKB has approval from the North West Multicentre Research Ethics Committee as a Research Tissue Bank approval.

## Results

### Study Population

The population characteristics of the contributing sites are presented in Table 1. The 39,976 participants covered a wide age range, from age 20 to 96 years, and 18,922 (47.3%) were men. Most participants were of European ancestry (97.2%), but this study also included persons from 2 Asians populations, and in the UKB, 1.8% self-reported non-Europeans were included.

The overall prevalence of PVS was 98%, while region-specific prevalence estimates were 59% (mesencephalon), 90% (hippocampus), 92% (basal ganglia), and 95% (centrum semiovale). When excluding automated procedures, the overall prevalence of PVS was 90% with region-specific estimates being considerably lower, namely 52% (mesencephalon), 69% (hippocampus), 64% (basal ganglia), and 73% (centrum semiovale) (eTable 3, links.lww.com/WNL/C400).

### Demographics

**Figure 2:**
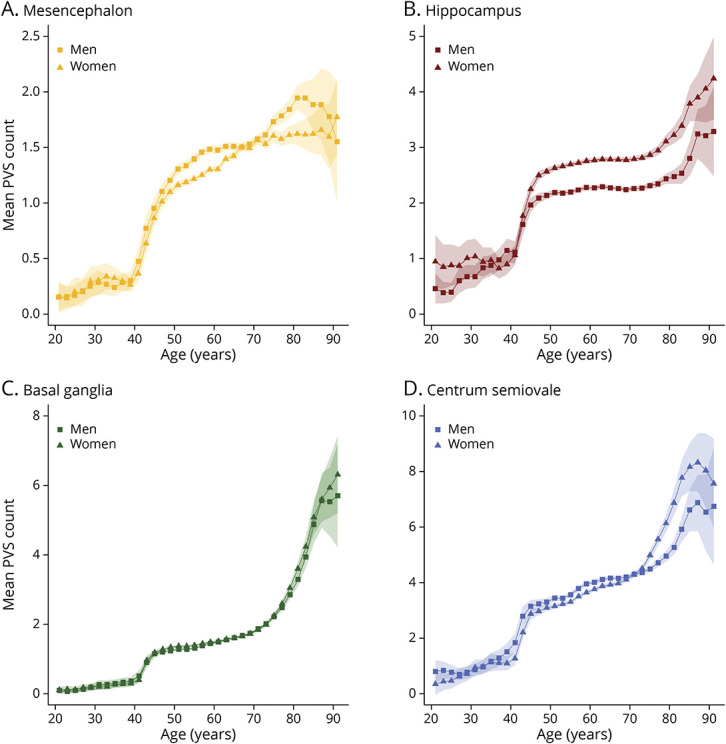
Age-Specific and Sex-Specific Trends of the Number of PVS in the 4 Brain Regions Mean counts of PVS across the lifespan in the 4 brain regions: mesencephalon (A), hippocampus (B), basal ganglia (C), and centrum semiovale (D). Mean PVS counts for men are depicted with squares, and mean PVS counts for women are depicted with triangles. Abbreviations: PVS = perivascular spaces.

**Figure 3:**
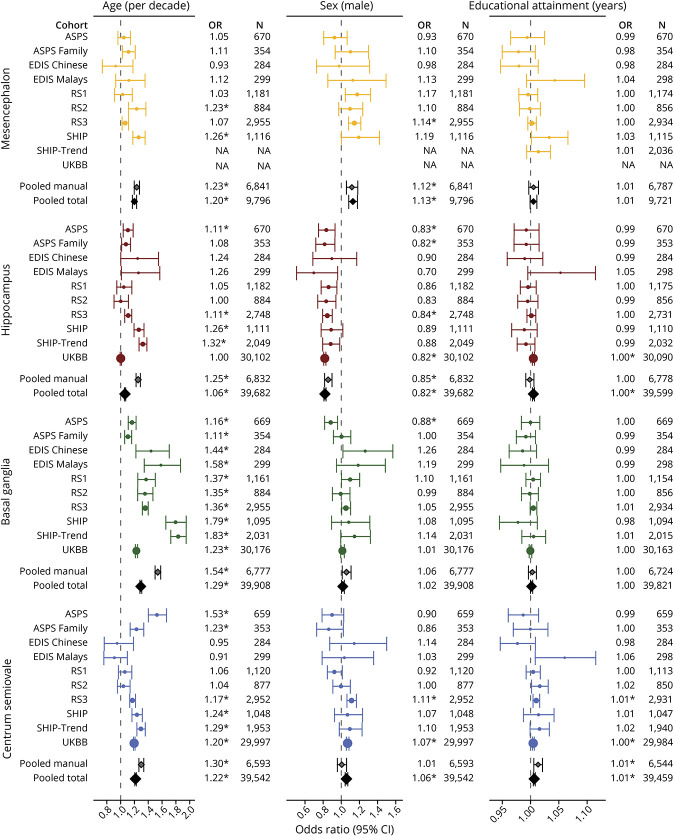
Study-Specific and Pooled Associations Between Demographic Factors and the Number of PVS in the 4 Brain Regions Forest plot showing odds ratios with corresponding 95% confidence intervals for the association between demographic factors and PVS counts in the 4 brain regions, both from 10 individual sites and pooled analyses. *Survived multiple testing correction (*p* < 0.05/19). Abbreviations: PVS = perivascular spaces.

First, we investigated demographic factors in relation to PVS counts. Figure 2 shows the age-specific and sex-specific trends of PVS counts in the 4 brain regions. PVS counts for most regions were higher within the manually rated data sets except for the mesencephalon (eFigure 1, links.lww.com/WNL/C398). Higher age was associated with more PVS in all regions, with the largest effect in the basal ganglia (OR per decade [95% CI] = 1.29 [1.28–1.30]) compared with the other regions (ORs between 1.06 and 1.22) (eTable 4, links.lww.com/WNL/C400). Men had more PVS in mesencephalon (1.13 [1.08–1.18]), centrum semiovale (1.06 [1.04–1.07]), and basal ganglia (1.02 [1.00–1.03]), whereas they had less in the hippocampus (0.82 [0.81–0.83]), particularly at older age. Years of education were associated with more PVS in the centrum semiovale (1.01 [1.00–1.01]) and hippocampus (1.00 [1.00–1.01]). Forest plots with all effect estimates, that is, per individual site and pooled, are shown in Figure 3.

### Cardiovascular Risk Factors

**Figure 4:**
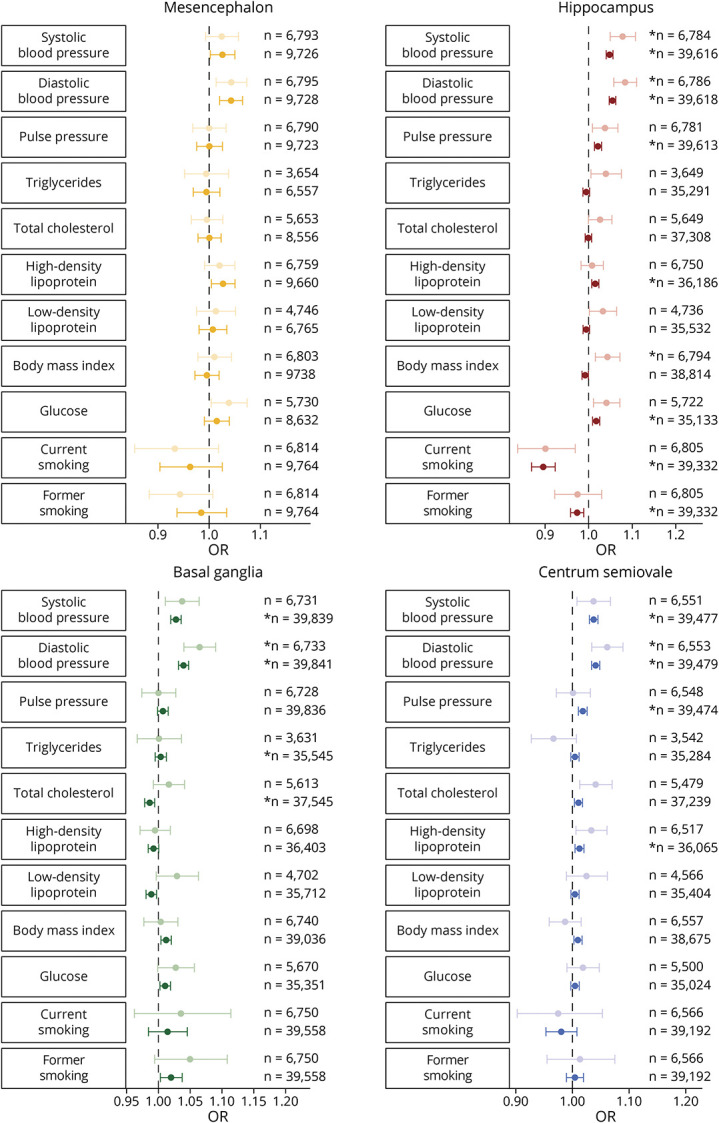
Pooled Associations Between Cardiovascular Risk Factors and the Number of PVS in the 4 Brain Regions Forest plot showing odds ratios with corresponding 95% confidence intervals for the association between cardiovascular risk factors and PVS counts in the 4 brain regions. Lighter colors correspond to pooled analyses including manual ratings only, and darker colors correspond to pooled analyses including both the manual and automated ratings. *Survived multiple testing correction (*p* < 0.05/19)*.* Abbreviations: PVS = perivascular spaces.

Next, we studied the relation between cardiovascular risk factors and PVS counts (Figure 4; eFigure 2, links.lww.com/WNL/C398; eTable 4, links.lww.com/WNL/C400). Higher BP was associated with more PVS in all regions, with the largest effects for diastolic BP. For the other cardiovascular risk factors, the significant associations were mostly for HDL cholesterol (hippocampus, 1.02 [1.01–1.02]; mesencephalon, 1.03 [1.00–1.05]; centrum semiovale, 1.01 [1.01–1.02]) and glucose levels (hippocampus, 1.02 [1.01–1.03]; basal ganglia, 1.01 [1.00–1.02]). Furthermore, there was an association between higher levels of total cholesterol and centrum semiovale PVS (1.01 [1.00–1.02]) and between a higher BMI and PVS in the basal ganglia (1.01 [1.00–1.02]) and centrum semiovale (1.01 [1.00–1.02]). Current smoking was related to lower counts of hippocampus PVS (0.90 [0.87 -0.93]). Former smoking however showed an association with lower counts of hippocampus PVS (0.97 [0.96 -0.99]) but higher counts within the basal ganglia PVS (1.02 [1.00 -1.04]).

After additional adjustment for other cardiovascular risk factors, the association with diastolic BP remained significant (eFigure 3, links.lww.com/WNL/C398; eTable 4, links.lww.com/WNL/C400).

### *APOE* Genotypes

**Figure 5:**
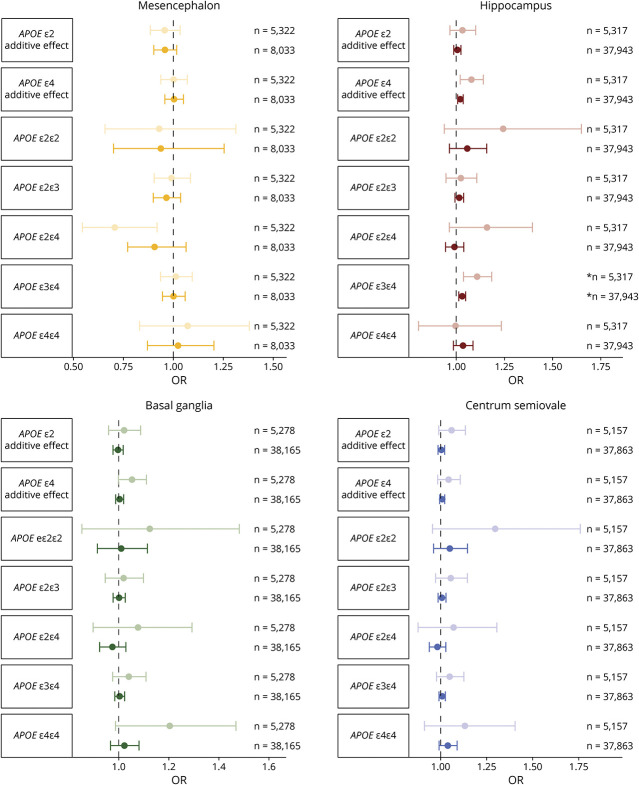
Pooled Associations Between *APOE* Genotypes and the Number of PVS in the 4 Brain Regions Forest plot showing odds ratios with corresponding 95% confidence intervals for the association between *APOE* genotypes and PVS counts in the 4 brain regions. Lighter colors correspond to pooled analyses including manual ratings only, and darker colors correspond to pooled analyses including both the manual and automated ratings. *Survived multiple testing correction (*p* < 0.05/19). Abbreviations: PVS = perivascular spaces.

We also investigated the effect of *APOE* genotypes on PVS counts (Figure 5; eFigure 4, links.lww.com/WNL/C398; eTable 4, links.lww.com/WNL/C400). The most significant association was identified for ε3/ε4 carriers and hippocampal PVS (1.03 [1.01–1.05]). Furthermore, there was a dose-dependent effect between ε4-alleles and hippocampus PVS (1.02 [1.01–1.04] per allele), but this was not significant for the other regions. Similar effect estimates were found after adjustment for cardiovascular risk factors (eFigure 5).

### MRI Markers

**Figure 6:**
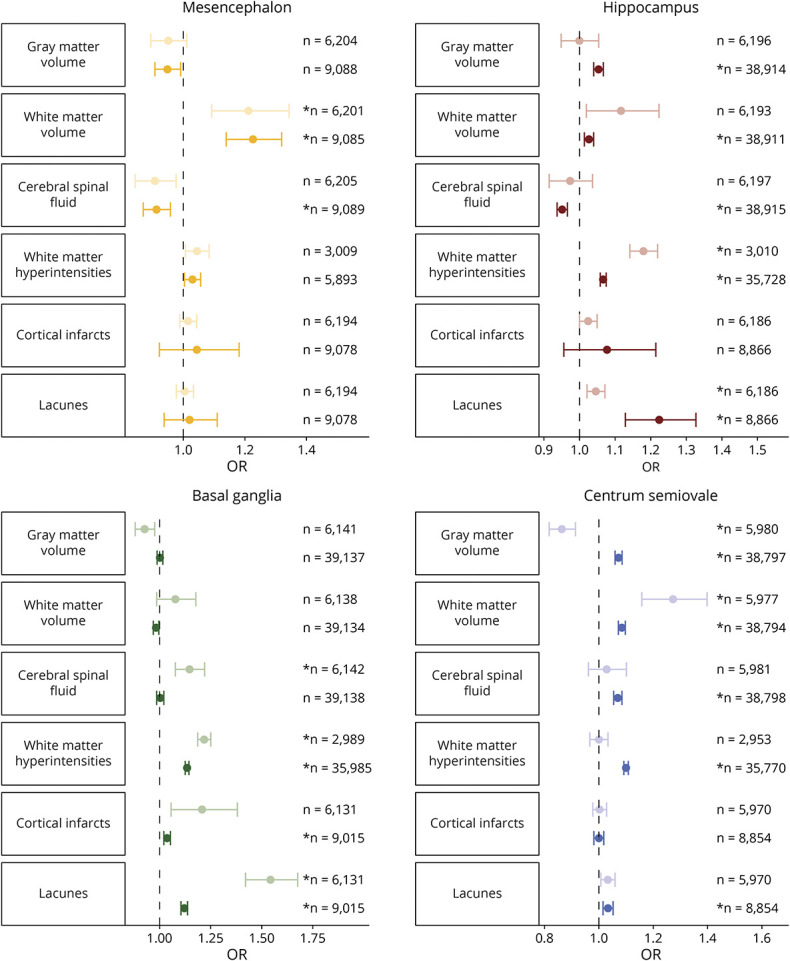
Pooled Associations Between MRI Markers and the Number of PVS in the 4 Brain Regions Forest plot showing odds ratios with corresponding 95% confidence intervals for the association between MRI markers and PVS counts in the 4 brain regions. Lighter colors correspond to pooled analyses including manual ratings only, and darker colors correspond to pooled analyses including both the manual and automated ratings. * Survived multiple testing correction (p < 0.05/19). PVS = perivascular spaces

Finally, we explored MRI markers in relation to PVS counts (Figure 6; eFigure 6, links.lww.com/WNL/C398; eTable 4, links.lww.com/WNL/C400). WMH volume and presence of lacunes were both associated with more PVS in multiple brain regions, with the strongest effects for the basal ganglia (1.14 [1.13–1.14] and 1.10 [1.09–1.12], respectively). The presence of cortical infarcts were also associated with more basal ganglia PVS (1.04 [1.02–1.05]). Further associations were present between larger gray matter volume and more PVS in the hippocampus (1.05 [1.04–1.07]) and centrum semiovale (1.07 [1.06–1.09]). For WM, larger volumes were associated with less basal ganglia PVS (0.98 [0.97–1.00]) and more PVS in the other regions, particularly in the mesencephalon (1.23 [1.14–1.32]).

After additional adjustment for cardiovascular risk factors, most associations remained similar or were slightly strengthened (eFigure 7, links.lww.com/WNL/C398; eTable 4, links.lww.com/WNL/C400). When also including other MRI markers in the model, most effects diminished except for the association between WMH and PVS in the hippocampus (1.16 [1.13–1.20]) and basal ganglia (1.17 [1.15–1.20]) and between WM and basal ganglia PVS (0.95 [0.92–0.98]) (eFigure 8; eTable 4). It is of interest that the direction of effect changed for the association between WM volume and centrum semiovale PVS (0.96 [0.93–1.00]).

### Sensitivity Analyses

When only including studies with manual PVS counts, most effect estimates were stronger (Figures 3–6; eTable 4, links.lww.com/WNL/C400). Some relationships even displayed opposite effects, such as the association between gray matter volume and centrum semiovale PVS (manual only 0.86 [0.82–0.91] and total pooled 1.07 [1.06–1.09]). These opposite effects were also seen between BMI and hippocampus PVS (manual only 1.04 [1.02–1.07] and total pooled 0.99 [0.98–0.99]). Differences in prevalence were also seen, with little variation across different ages within the automated quantification set (eFigure 1, links.lww.com/WNL/C398). Associations between individuals older than 60 years and younger than 60 years did not differ substantially (eFigure 9).

When comparing prevalence across ethnicities, higher PVS counts in the hippocampus and centrum semiovale were observed for participants with an African ethnicity, particularly in the age stratum of 60 years and older (eFigure 10, links.lww.com/WNL/C398). For most relationships between determinants and PVS counts, the results did not differ significantly (eFigure 11). Differences were however observed for the association between sex and centrum semiovale PVS, with higher PVS counts for men in the European and Asian subgroups, yet lower counts in the African subgroup (European 1.07 [1.06–1.09], African 0.81 [0.68–0.96], and Asian 1.06 [0.93–1.19]). Further differences were seen for the associations of *APOE* genotypes, with significant associations between ε4/ε4 carriers and centrum semiovale PVS and ε4-allele dosages and hippocampus PVS observed in the Asian group only (2.24 [1.25–4.01] and 1.17 [1.01–1.36], respectively).

## Discussion

PVS have been hypothesized to enlarge in response to various brain pathologies. Studies on the determinants of PVS have been limited to relatively small single-site investigations and use widely different methodologies, making it difficult to disentangle true associations from false and/or biased results. The results of our multisite analysis of population-based cohort studies showcase this strikingly, with variable results from individual sites converging into a reliable overall picture of PVS determinants. We found that increasing age was related to more PVS throughout the brain, while other determinants were region-specific, including sex, cardiovascular risk factors, *APOE* genotypes, and MRI markers of CSVD. Our results support the notion that PVS have a multifactorial origin and highlight the power of collaborative efforts.

Of all investigated factors, age was among the most important PVS determinants. Although PVS could be seen even in the youngest participants in their early twenties, a sharp increase in PVS counts was apparent from age 40 years, particularly within manually rated data. The prevalence of PVS in the various regions ranged between 7.5% and 98% depending on the age group and between 47% and 100% across the whole brain. Three other population-based studies have reported a wide variety of prevalences, namely 16% (mean age 76 years),^41^ 87.5% (mean age 72 years),^3^ and 100% (mean age 73 years).^1^ These large differences likely reflect heterogeneity in methods, ie, rating scales and factors related to the MRI scanner, such as field strength, sequence, and image resolution. Different thresholds for PVS sizes were also defined,^1,3,41^ resulting in higher estimates for high-resolution images.^1^ We found that the effect of age was strongest for the basal ganglia (OR per decade = 1.29) compared with the other regions (ORs 1.06–1.22), representing a striking difference of PVS increase across regions within the same period. This is in line with a mouse study and a meta-analysis of cohort studies that found regional differences of PVS in response to aging.^2,8^ This indicates that, rather than a shared process leading to more PVS throughout the brain, there might be factors contributing specifically to pathology in certain regions.

One striking region-specific factor was sex, for which we report several novel findings. Men had more PVS in the mesencephalon, a region where sex differences have been described with respect to both its structure and function.^42^ The mesencephalon is important for motor control and cognition, but PVS have remained understudied and are mostly the subject of case reports. One study did not find an association of sex with mesencephalon PVS, but pooled all infratentorial regions together.^3^ In that study, women had higher subcortical WM PVS scores, whereas a study in Chinese stroke patients reported higher scores in men.^15^ We found significant sex differences for the centrum semiovale, with more PVS in men, in line with a previous study that found higher WM PVS volumes for.^43^ By contrast, more hippocampal PVS were seen in women, as observed earlier.^15^ We did not identify any sex differences in the basal ganglia, in line with most previous studies,^3,5,15^ but contrary to 1 study that found more PVS in men.^1^ The sex differences could be due to differences in brain development, but comparisons between men and women of the morphological and functional aspects of PVS remain to be reported. In addition, the differences were most apparent later in life, suggesting a differential susceptibility to age-related brain pathologies. In light of women's higher risk of Alzheimer disease, it is interesting that they have more hippocampus PVS and to a lesser extent centrum semiovale PVS because amyloid-β is disproportionately deposited in hippocampal and cortical tissues.^44,45^ Another remarkable finding is the negative association between smoking and hippocampus PVS counts. Previous studies were inconclusive for this relation but suggested no association.^4^ Therefore, further research is needed to replicate this finding and explore possible underlying pathophysiologic mechanisms.

We also found that a higher systolic, and particularly diastolic, BP was associated with more PVS. High BP has also been related to other CSVD markers, including WMH, infarcts, and microbleeds; this included reports of differential associations between systolic and diastolic BP.^46,47^ The stronger associations with diastolic pressure, rather than systolic, suggest that the lower bound of BP is more important for PVS enlargements. A possible explanation is that a continuously raised diastolic BP leads to a greater extravasation of fluid into the perivascular space or alternatively prevents sufficient fluid from returning into the bloodstream (after a systolic pulse). Gutierrez et al.^3^ suggested that PVS might arise behind a large drop in vascular caliber that exposes the smaller vessels to greater pulsatility and mechanical forces, which is the case for arteries in the basal ganglia and brainstem. Although previous research has reported high BP and hypertension^8^ as a determinant of PVS severity in the basal ganglia,^3,16^ our novel finding with mesencephalon PVS provides further support for this hypothesis. However, pulse pressure, as a measure of the pulsatile component of BP, was not strongly related to PVS counts. Future studies should use more extensive ways of measuring the compliance and distensibility of arteries, preferably in vessel beds relevant for the brain.

Most cardiovascular risk factors associations were strongest for hippocampal PVS. There is some debate on whether these fluid-filled cavities in the hippocampus actually represent PVS. Some define these lesions as hippocampal sulcal cavities that are believed to be a remnant from brain development.^48^ Furthermore, it has recently been suggested that a subset of these, which seem hyperintense on FLAIR, might actually represent microinfarcts.^18^ Nevertheless, others have observed characteristics of typical PVS, namely the presence of a vessel within these lesions that is surrounded by a fluid-filled compartment without apparent damage to the surrounding tissue.^48^ Our finding that cardiovascular risk was related to hippocampus PVS supports this potential vascular origin. Another finding that could add to this is the link with *APOE* ε4 genotypes, which influences lipid metabolism and increases risk of cardiovascular disease.^49^ However, *APOE* ε4 is also an important risk factor for Alzheimer disease and predisposes to amyloid pathology in the brain, particularly in the hippocampus.^50^ Furthermore, *APOE* ε4 may disrupt perivascular drainage of soluble amyloid-β from the brain and thereby increase the risk for Alzheimer disease.^e1^ It remains to be determined whether the association between *APOE* ε4 and hippocampal PVS reflects a cardiovascular or amyloid-related pathway or both.

The link between WMH and basal ganglia PVS has been extensively described,^5,8,17^ and our study confirms this. The mechanism underlying this association has not been elucidated, but a possible explanation is that drainage from the (periventricular) WM goes through the basal ganglia PVS. Alternatively, shared determinants could induce an association, but there was little influence by additional adjustment for cardiovascular risk factors and other MRI markers. Nonetheless, potential shared factors not assessed in this study, such as genetics, could well play a role. For lacunes, another consideration is misclassification as PVS or vice versa. However, we paid particular attention to differentiating PVS from lacunes using their shape, size, and presence of a hyperintense rim on FLAIR images.^27^ Furthermore, lacunes are per definition different lesions because PVS larger than 3 mm were rated separately in our rating protocol,^27^ and this is also the lower size bound of lacunes. Nevertheless, it remains possible that the presence of lacunes might have influenced the counting of PVS, but these visual ratings cannot be performed in a blinded fashion. However, we also find various associations with other MRI markers, including larger CSF and basal ganglia, hippocampus and centrum semiovale PVS, as well as smaller gray matter volume and centrum semiovale PVS when adjusting for other cardiovascular and MRI markers, suggesting a relation with tissue loss. The PVS could arise as part of the neurodegenerative process, for example, through insufficient clearance of neurotoxic proteins, but another explanation is that they simply become visible as a secondary consequence of neurodegeneration by filling up the empty space created by brain atrophy. However, multiple relationships with larger tissue volumes and PVS across regions were seen. Longitudinal studies are required to determine the temporal relation between PVS and brain atrophy.

Research investigating ethnic differences in CSVD markers has reported differential effects in relation to lacunes and WMH.^e2,e3^ However most studies found no differential effects in relation to microbleeds,^e4-e6^ and thus, far little exploration of ethnic differences has been performed in relation to PVS.^8^ One previous study did find both basal ganglia and centrum semiovale PVS to be more prevalent in White than Chinese patients who had had a transient ischemic attack or stroke.^9^ Another study in stroke-free individuals found higher overall PVS scores in Black individuals compared to White,^3^ in line with our findings for African individuals. This study also reveals some ethnic differences in the association between determinants and PVS counts, such as for sex and *APOE* ε4/ε4 carriership, mostly within the centrum semiovale. Differential effects of *APOE* alleles across ethnicities have been described previously in the context of Alzheimer disease biomarkers^e7,e8^ and intracerebral hemorrhage^e9^ but have to our knowledge not yet been described for PVS. Despite these ethnic differences in this study, the included non-European samples were derived from a European-based data set, non-European groups were small, and not all ethnicities were represented. Therefore, more research into larger samples of non-European individuals is needed to unravel whether mechanisms truly differ across ethnicities.

Although the automated method used in UKB and RS3 was based on the manual rating method, this difference in classification may have affected the results. When excluding automated methods, the effects between determinants and PVS counts became stronger. This may partly be due to differences in population characteristics, particularly in the UKB given the previously described “healthy volunteer bias,”^e10^ which may have resulted in an underestimation of the effects. It may also have been affected by the use of T1-weighted images within which PVS are less visible than on T2-weighted images. However, this difference in the magnitude of effect was also seen for the association between lacunes and basal ganglia PVS—an association that could not be tested in the UKB because this cohort was missing lacune information. In fact, when excluding only RS3 from the pooled analysis, the association increased 3-fold.^28,29^ These observations suggest that the automated method causes a dilution of the results, despite the automated method reporting high reliability and reproducibility metrics.^28,29^ Nevertheless, the classification method should be taken into consideration when interpreting results.

Strengths of this study include the large sample size resulting from a multisite effort; the rigorous harmonization of rating protocols, including a minimal size criterion, allowing data pooling; the use of continuous measures (PVS counts) instead of categorization (i.e. grades/severity scales); appropriate statistical handling with negative binomial regression models; and the investigation of 4 different brain regions, resulting in several region-specific associations. In addition, sensitivity analyses through ethnicity have not been performed within the current PVS literature. This study also has several limitations. One possible limitation is that for the 2 larger brain regions, where hundreds of PVS can be present, only a single slice was used for rating to reduce the time needed for PVS counting. However, we have previously shown that this is sufficient to capture the burden across the whole region, with high correlations between single-slice and whole-region approach.^30^ Furthermore, the mesencephalon was not rated using the automated method within the UKB because of its unreliability using T1-weighted images. In addition, within the age trends, the use of summed PVS counts across the 4 regions may not fully capture total brain PVS. Further methods need to be developed to reliably extract this metric. The use of multiple MRI scanners and protocols is strength because it validates the findings beyond a single setup but also a limitation because it introduces an additional source of variability. Furthermore, although this is the largest study on PVS to date, we potentially did not have enough power to explore all ethnic differences because of the limited non-European samples. Finally, the cross-sectional study design does not inform whether the determinants precede PVS development or the other way around.

Although the focus of this study was on cardiovascular risk factors and MRI markers of CSVD, it would be interesting for future studies to investigate other potential determinants to further disentangle potential differences in etiology of PVS per region, including lesser investigated regions such as the mesencephalon. These include MRI markers, such as microbleed locations, WM microstructure, and functional MRI. In addition, it is possible that PVS reflect a more systemic pathology. We and others have shown links with the retinal microvasculature,^e11^ kidney function,^e12^ and inflammation,^23,e13^ but these findings have yet to be replicated in large studies. Furthermore, although recent studies and meta-analysis are shedding light on the relationship between PVS and clinical outcomes, such as cognitive decline,^7,8,35,e14,e15^ stroke,^7,8^ and dementia,^7,e15,e16^ the results are inconclusive and larger samples with harmonized classification methods are needed.

In conclusion, factors related PVS enlargement include age, sex, cardiovascular risk factors, *APOE*, and other MRI markers. There seems to be important regional specificity for these associations, potentially reflecting heterogeneity in etiology.

## Glossary

ASPS: Austrian Stroke Prevention Study
BMI: body mass index
CSVD: cerebral small vessel disease
EDIS: Epidemiology of Dementia in Singapore
FLAIR: fluid-attenuated inversion recovery
HDL: high-density lipoproteins
ICC: intraclass correlation coefficient
ICV: intracranial volume
PVS: perivascular spaces
RS: Rotterdam Study
SHIP: Study of Health in Pomerania
UKB: UK Biobank
WM: white matter
WMH: WM hyperintensity
